# Improving recruitment to clinical trials with a register of a million patients who agree to the use of their clinical records for research in the Scottish Health Research Register (SHARE)

**DOI:** 10.1186/1745-6215-12-S1-A115

**Published:** 2011-12-13

**Authors:** Frank M Sullivan, Shaun Treweek, Anile Grant, Fergus Daly, Donald Nicolson, Brian McKinstry, Janet Hanley, Jenny Ure, Aziz Sheikh

**Affiliations:** 1 Division of Population Health Sciences, The University of Dundee DD2 4BF, UK; 2 Centre for Population Health Sciences, The University of Edinburgh EH8 9AG, UK

## Background

The UK’s technical ability to identify people eligible for medical research is not yet matched by a practical capability to approach them directly to ask them to consider participation in those studies. The consequence is that recruitment to research is more difficult than necessary and some projects fail. This makes Britain a less attractive location to undertake clinical research than it should be. In order to overcome this increasingly important obstacle, we wanted to develop a register of Scottish residents who wish to be considered for participation in a range of studies.

Current legal and research governance framework prevents researchers from making direct contact with potentially eligible subjects; rather, the initial contact must come through a clinician who has direct responsibility for their care. Two exceptions to this are where researchers advertise the existence of a study to the whole population e.g. through the mass media as was the case in UKBiobank or for 20 000 patients with diabetes mellitus registered on Scottish Care Information Diabetes Collaboration who have agreed to be approached directly via the Scottish Diabetes Research Network (SDRN). If sufficient numbers of people in Scotland would consider registering to participate in research, this resource could be extended to a wide range of researchers.

## Methods

Deterministic record linkage using the Community Health Index number allows linkage to a wide variety of data sources, this enables the identification of people on the basis of their demographic, diagnostic and therapeutic characteristics. This capability already enables the use of clinical records for epidemiological studies (using anonymised data) or follow-up of study subjects who have participated in clinical trials. We have completed qualitative work (nine focus groups and 17 interviews with patients, clinicians and researchers) to specify the features of the register which has been developed and are initiating a nationwide recruitment process to enrol one million Scottish residents.

## Results

All groups of respondents were supportive of the plans, the technical implementation was successful and recruitment has proven feasible. 13% of people approached in a single letter from their GP agreed to participate as shown in the figure.

**Figure 1:**
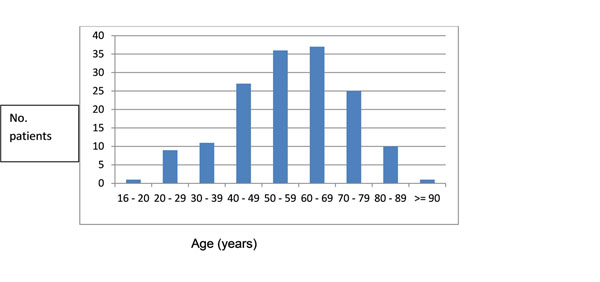


## Conclusions

We suggest that the public should be supported in moving away from passively waiting for an approach about an interesting piece of health research towards being able to express an interest in participation in health research. Facilitating this active approach to increase the efficiency of clinical research in Scotland is the purpose of SHARE.

